# Electroluminescence From a 1D Metal–Organic Chalcogenide Enabled by a Minute‐Scale Facile Synthesis

**DOI:** 10.1002/advs.202513328

**Published:** 2025-09-24

**Authors:** Sang‐Hyun Chin, Daseul Lee, Donggyu Lee, Seunghwan Kim, Byeongjoo Kang, Kwanghyun Chung, Tong‐Il Kim, Jieun Yeon, Su Hwan Lee, Sang Woo Bae, Woojae Kim, Soohyung Park, Kwanpyo Kim, Young‐Hoon Kim, Yeonjin Yi

**Affiliations:** ^1^ Department of Physics Yonsei University Seoul 03722 Republic of Korea; ^2^ Department of Energy Engineering Hanyang University Seoul 04763 Republic of Korea; ^3^ Advanced Analysis Center Korea Institute of Science and Technology (KIST) Seoul 02792 Republic of Korea; ^4^ Department of Chemistry Yonsei University Seoul 03722 Republic of Korea; ^5^ Division of Nano & Information Technology KIST School University of Science and Technology (UST) Seoul 02792 Republic of Korea

**Keywords:** electroluminescence, electronic structures, metal–organic chalcogenides, synthesis, vapor deposition

## Abstract

Metal–organic chalcogenides (MOCs) represent a unique materials platform promising to overcome the respective stability and structural integrity challenges of perovskites and functionalized dichalcogenides. However, their practical application is hindered by slow, multi‐day synthesis methods that produce low‐quality films. Here, these challenges are addressed with a vapor‐assisted solution process that enables the ambient‐pressure fabrication of 1D MOC, silver(I) 2‐methyl ester benzenethiolate (AgSPhCOOMe), films within 5 min. The resulting dense, pinhole‐free AgSPhCOOMe films exhibit a high photoluminescence quantum yield of 37.5%, with bright, broadband emission originating from self‐trapped excitons due to the material's strong electron‐phonon coupling. This scalable synthesis platform enables the successful integration of these MOCs into light‐emitting diodes, demonstrating electroluminescence from this material class. By engineering the charge‐transport layers to achieve balanced injection, a maximum external quantum efficiency of ≈0.1% is achieved. The in situ photoelectron spectroscopy analysis reveals that a significant electron injection barrier (0.62 eV) remains even in the optimized device, identifying this as the main efficiency bottleneck. Therefore, this work provides a foundational platform for MOC‐based devices and a clear roadmap focused on new ligands and interface engineering to realize their full potential as high‐performance, solution‐processable emitters.

## Graphical abstract

From Minutes to Light: A rapid, vapor‐assisted solution process of highly luminescent 1D metal–organic chalcogenide (MOC) films enables the fabrication of the first MOC‐based LEDs, unlocking electroluminescence from this emerging class of materials.

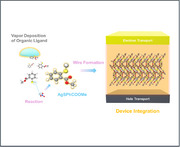

## 1 Introduction

The development of next‐generation electronic and optoelectronic devices has driven extensive research into low‐dimensional materials, such as graphene and transition metal dichalcogenides (TMDCs).^[^ ^1^ ^]^ A common strategy to tailor their physicochemical properties involves introducing heterogeneous elements or organic molecules onto their surfaces. However, this post‐synthesis functionalization often requires harsh conditions that can compromise the structural integrity of the nanosheets and typically results in a non‐uniform distribution of functional groups.^[^ ^2^ ^]^ These materials often lack a well‐defined atomic structure, which complicates efforts to establish clear structure‐property relationships, hindering their widespread application.

In parallel, low‐dimensional halide perovskites have emerged as exceptionally promising candidates for optoelectronics, particularly for light‐emitting applications. By incorporating bulky organic cations (L) into the 3D ABX_3_ — where A is a monovalent cation, either an organic or an inorganic, B is a divalent heavy metal ion, and X is a halide anion — crystal structure, quasi‐2D phases (L_2_[ABX_3_]_(n–1)_BX_4_) are formed. These materials exhibit increased exciton binding energies (*E_b_*) and reduced trap densities, which are highly favorable for efficient radiative recombination.^[^ ^3^, ^4^, ^5^, ^6^, ^7^, ^8^, ^9^, ^10^, ^11^ ^]^ Despite their impressive performance, the practical viability of halide perovskites is fundamentally limited by their poor intrinsic stability, which originates from the ionic nature of their chemical bonds.^[^ ^12^ ^]^

Metal–organic chalcogenides (MOCs), first reported in 1991, offer a promising solution to these challenges.^[^ ^13^ ^]^ Unlike conventional low‐dimensional materials, MOCs feature a unique hybrid structure where inorganic metal chalcogenide units are covalently bonded to organic ligands, forming frameworks that can range from 1D wires and 2D sheets to 3D networks.^[^ ^14^, ^15^, ^16^ ^]^ This structure promises a combination of stability and tunable functionality. The potential of MOCs as light emitters was first highlighted in 2018 with the synthesis of 2D silver benzene‐selenolate ([AgSePh]_∞_), or “mithrene”.^[^ ^14^ ^]^ However, early methods were hindered by impractically long reaction times (3–10 days) and produced precipitates with morphologies unsuitable for thin‐film devices. Subsequent efforts to create device‐compatible films by tarnishing silver with organochalcogen vapor were also slow (3 days) and resulted in a low photoluminescent quantum yield (PLQY) below 0.1% at room temperature.^[^ ^17^ ^]^ While a recent breakthrough demonstrated a highly luminescent 1D MOC with a PLQY of 22%, its synthesis still required a lengthy 5‐day reaction.^[^ ^16^ ^]^ Thus, despite progress in achieving desirable photoluminescence (P), the dual challenges of slow synthesis and the absence of a viable path to device integration have persisted.^[^ ^18^, ^19^, ^20^, ^21^ ^]^ Critically, a key milestone for this material class remains elusive: to date, electroluminescence from any MOC‐based device has not been reported.

In this study, we address the ongoing challenges associated with the production of high‐quality MOC films by developing a rapid, vacuum‐free synthetic route. This enables the first integration of MOC films into electroluminescent devices. We employ a facile chemical vapor deposition method known as vapor‐assisted solution processing (VSP), which has previously been utilized for halide perovskite fabrication.^[^ ^22^, ^23^ ^]^ The key innovation that distinguishes our approach from previous MOC synthesis, which still relied on vacuum‐deposited metal films,^[^ ^24^, ^25^ ^]^ is the strategic combination of a solution‐processable inorganic precursor (silver(I) nitrate) and an ambient‐pressure sublimable organic ligand (methyl thiosalicylate, denoted as HSPhCOOMe). This combination facilitates a simple two‐step process involving spin‐coating, followed by a brief vapor reaction. Using this method, we demonstrate the synthesis of 1D, wire‐structured MOC films and their successful integration into light‐emitting diodes (MOCLEDs), which achieve an external quantum efficiency of up to 0.1%.

## 2 Results and Discussion

**Figure 1:**
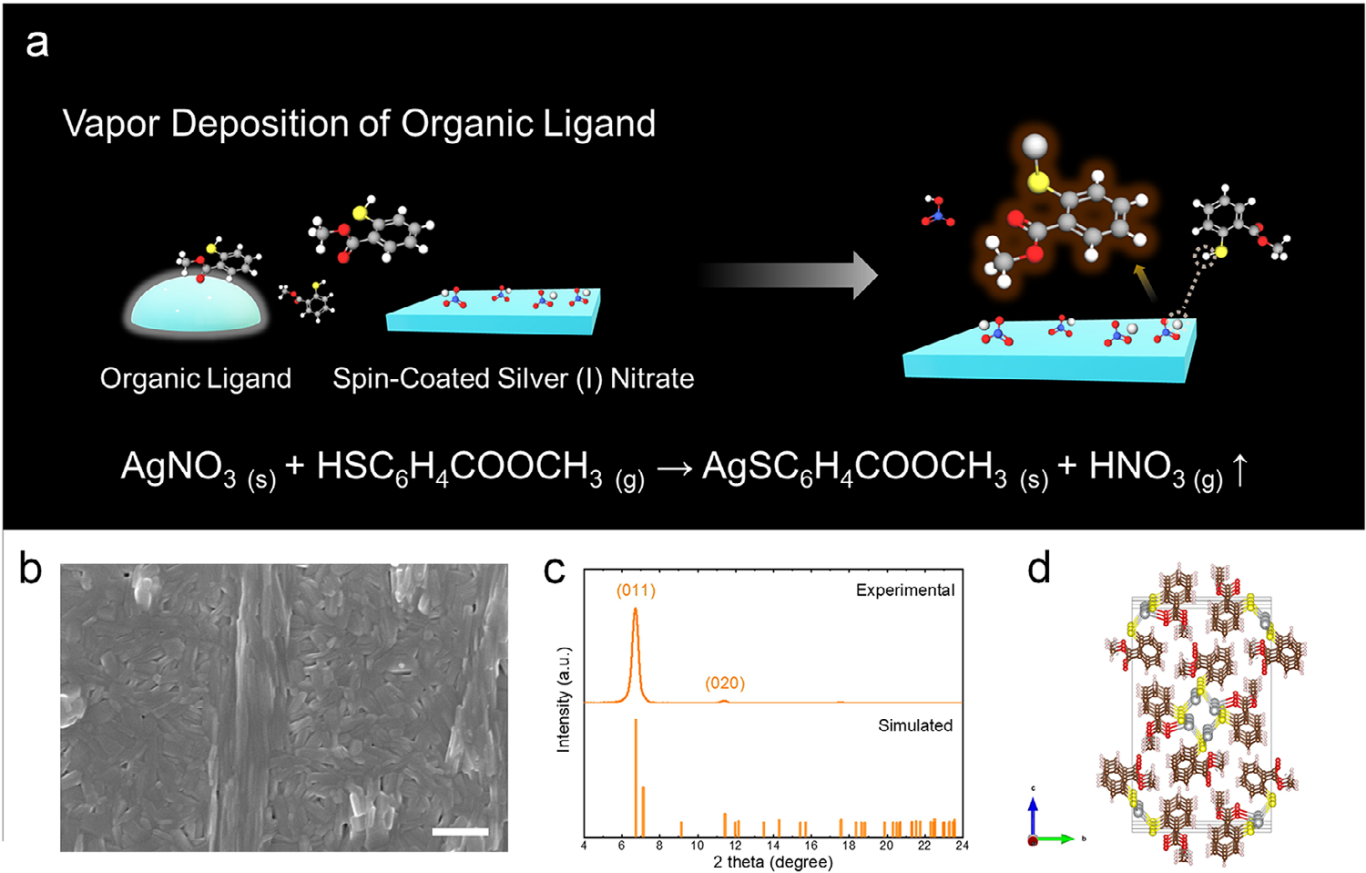
Synthesis and characterization of VSP‐grown AgSPhCOOMe films. a) Schematic of the vapor‐assisted solution process (VSP). b) Scanning electron microscopic (SEM) image of a resulting film (scale bar: 500 nm). c) Experimental X‐ray diffraction (XRD) pattern of the film compared with the simulated pattern derived from its crystallographic information file (CCDC‐2212139). d) Visualization of the 1D crystal structure.

The 1D MOC, silver(I) 2‐methyl ester benzenethiolate (AgSPhCOOMe), was synthesized using the VSP, as depicted in **Figure** **1**a. The synthesis began with the preparation of a precursor film by spin‐coating a 0.1 m silver(I) nitrate solution onto an indium tin oxide (ITO) substrate at 8000 rpm. This film was then placed in a sealed petri dish with liquid‐phase HSPhCOOMe and heated to 130 °C. During this process, the silver ion displaces the thiol proton to form the solid AgSPhCOOMe MOC and nitric acid as a byproduct, following the reaction:^[^ ^26^ ^]^ AgNO_3(s)_ + HSPhCOOMe_(g)_ → AgSPhCOOMe_(s)_ + HNO_3(g)_ ↑. The volatile nitric acid (boiling point: 83 °C) was released upon opening the petri dish, leaving the target film. The resulting AgSPhCOOMe films exhibit notable chemical robustness, showing no delamination and retaining their luminescence (under 365 nm excitation) when soaked in various common solvents such as water, acetonitrile, chlorobenzene, ethanol, and dimethyl sulfoxide. This chemical robustness indicates that the highly soluble silver(I) nitrate precursor has been fully converted into the insoluble MOC product, thereby confirming the completion of the reaction (Figure S1, Supporting Information).

The morphology and crystallographic properties of the synthesized films were then investigated. Figure 1b shows a scanning electron microscopy (SEM) image of a pinhole‐free, compact, polycrystalline film composed of long, rod‐like grains several hundred nanometers in length. To further investigate the film's topography and integrity, we performed atomic force microscopy (AFM) and cross‐sectional SEM (Figure S2, Supporting Information). The AFM imaging confirms a continuous, pinhole‐free film with full substrate coverage, exhibiting a root‐mean‐square (RMS) roughness of 11.049 nm, a value that directly reflects the rod‐like structure. This finding is corroborated by cross‐sectional SEM, which reveals a uniformly dense and compact layer, confirming the robust integrity of the film. This morphology is well‐suited for fabricating optoelectronic devices, and the film's structural properties were subsequently examined by X‐ray diffractometry (XRD), as shown in Figure 1c. Whereas multiple diffraction peaks are typically observed in previous reports,^[^ ^16^, ^26^ ^]^ our VSP‐grown film exhibits the exceptional dominance of the (011) peak, observed at 6.7°, with other reflections being suppressed. This result strongly indicates that our method produces a film with a significantly higher degree of preferential orientation, with the (011) planes aligned parallel to the substrate. This structural alignment is a unique characteristic of VSP‐grown films, distinguishing them from those prepared by other methods. The preferred alignment is further corroborated by the rod‐like morphology observed in AFM measurements and is consistent with the inherent 1D packing of the monoclinic AgSPhCOOMe crystal structure, as Figure 1d (CCDC‐2212139).^[^ ^26^ ^]^ To further investigate the local structural property, we additionally performed transmission electron microscopy (TEM) in Figure S3 (Supporting Information). High‐resolution imaging and selected area electron diffraction confirmed that the film is composed of highly crystalline, which is a key prerequisite for efficient optoelectronic performance, providing a comprehensive understanding of the film's crystallographic texture.

**Figure 2:**
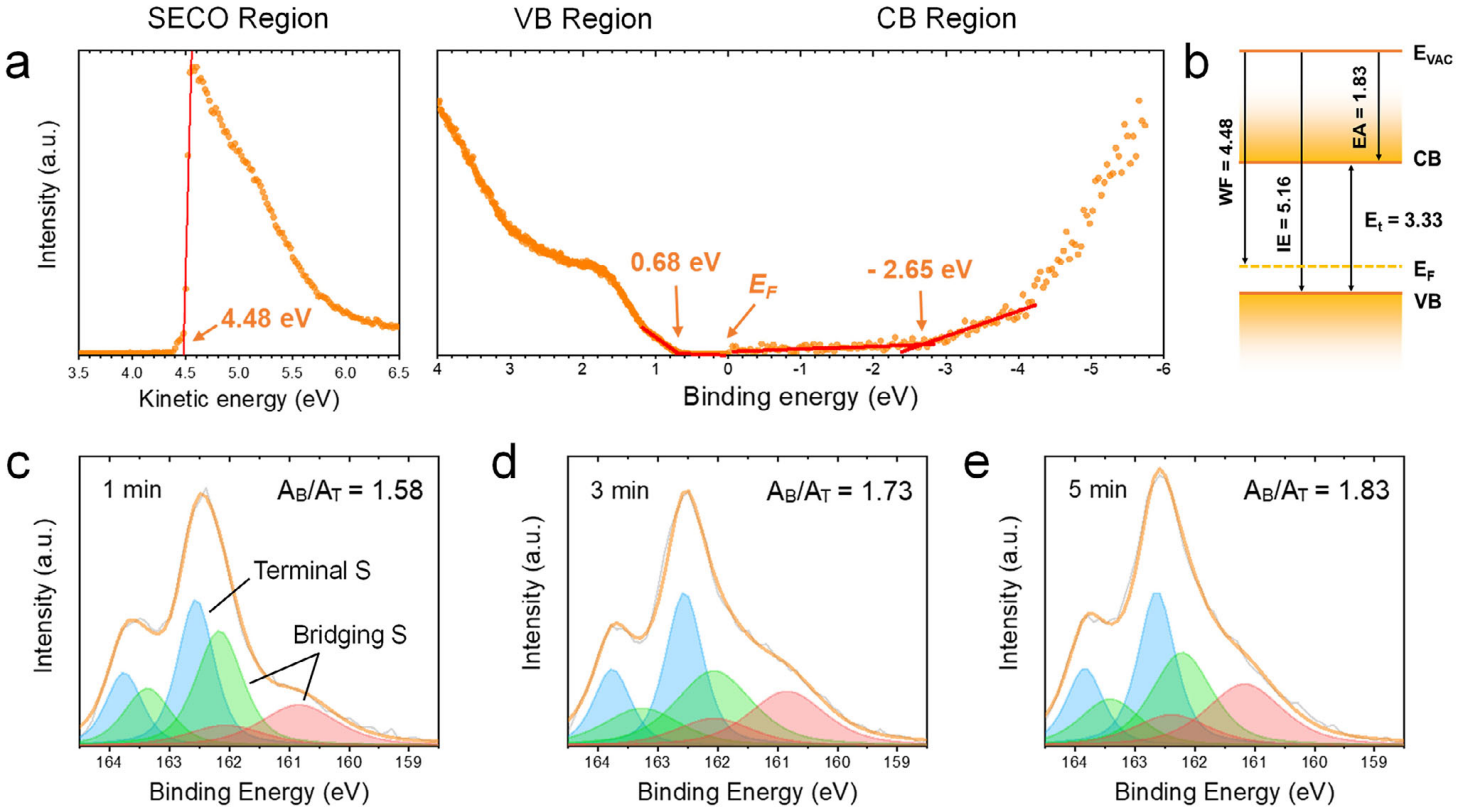
Electronic Structures and chemical bonding evolution of VSP‐grown AgSPhCOOMe films. a) UV photoelectron spectroscopy (UPS) and inverse photoemission spectroscopy (IPES) spectra of a fully reacted MOC film, showing secondary electron cutoff (SECO), valence band (VB), and conduction band (CB) regions. b) Energy‐level diagram derived from the spectra in (a). (**E_VAC_** and **E_F_** denote vacuum and Fermi level, respectively.) c–e) S 2p XPS spectra of MOC films prepared with varying reaction times (1, 3, and 5 min), showing the evolution of terminal (**A_T_**, blue) and bridging (**A_B_**, green and red) sulfur species.

Following the structural and morphological characterization, we investigated the electronic properties of the AgSPhCOOMe films to assess their suitability for optoelectronic applications. UV and inverse photoelectron spectroscopy (UPS/IPES) were employed to determine the electronic energy levels of the fully formed films (**Figure** **2**a). From these spectra, the work function (WF, 4.48 eV), ionization energy (IE, 5.16 eV), and electron affinity (EA, 1.83 eV) were evaluated, as summarized in the energy level diagram in Figure 2b. From the measurements, an electronic transport bandgap (E_t_) of 3.33 eV was determined. This value is in good agreement with the theoretically predicted value of 3.5 eV and indicates that the MOC has strong *p*‐type characteristics.^[^ ^16^ ^]^

To elucidate the mechanism behind the rapid VSP synthesis, we then monitored the chemical bonding evolution as a function of reaction time using X‐ray photoelectron spectroscopy (XPS). For sulfur compounds bonded to coinage metals, the S 2p binding energy is known to shift to lower values as its coordination number to the metal atoms increases, due to greater sharing of electron density.^[^ ^27^ ^]^ We therefore measured S 2p spectra for samples reacted for 1, 3, and 5 min (Figure 2c–e). The spectra were deconvoluted into components corresponding to two structurally distinct species: terminal sulfur (A_T_, ≈162.5 eV, blue), located at the ends of the 1D chains, and bridging sulfur (A_B_, ≈162.0 eV and ≈161 eV, green and red), which forms the internal backbone (detailed atomic structures are shown in Figure S4, Supporting Information). Notably, the area ratio of bridging‐to‐terminal sulfur (A_B_/A_T_) increases progressively from 1.58 (1 min) to 1.73 (3 min) and finally to 1.83 (5 min). This trend, where the proportion of internal backbone sulfur (A_B_) increases relative to the chain‐end sulfur (A_T_), provides direct spectroscopic evidence for the continuous elongation of the 1D AgSPhCOOMe chains. This highlights the fundamental role of bridging sulfur atoms in extending the 1D framework.^[^ ^16^, ^27^ ^]^

**Figure 3:**
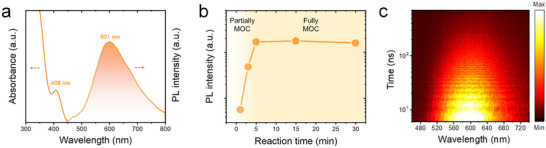
Optical Properties of VSP‐grown AgSPhCOOMe films. a) Absorbance and photoluminescent (PL) spectra. b) Evolution of the integrated PL intensity as a function of VSP reaction time. c) Time‐resolved PL intensity map showing emission as a function of wavelength and time after excitation by a 405 nm pulsed laser.

The optical properties of the VSP‐grown AgSPhCOOMe films were first characterized by steady‐state spectroscopy (**Figure** **3**a). The absorption spectrum exhibits a distinct excitonic transition peak (*E_exc_*) at 408 nm (3.04 eV). Comparing this with the *E_t_* (3.33 eV from Figure 2) reveals a large exciton binding energy (*E_b_*) of 290 meV,^[^ ^28^ ^]^ which is characteristic of strongly confined carriers in a low‐dimensional system. This strong carrier confinement suppresses non‐radiative decay pathways, thereby promoting efficient radiative recombination and resulting in a high photoluminescence quantum yield (PLQY) of 37.5% (Figure S5 and Table S1, Supporting Information). The PL spectrum exhibits defining features that are characteristic of emission from self‐trapped excitons (STEs): an exceptionally broad full width at half maximum (FWHM) of 165 nm and a notably large Stokes shift of 193 nm. Furthermore, the PL excitation‐emission map (Figure S6, Supporting Information) confirms that the emission profile is independent of the excitation wavelength. These characteristics are signatures of systems with strong electron‐phonon coupling, where the photoexcited carriers induce local lattice distortions to create a relaxed, lower‐energy emissive state.^[^ ^29^, ^30^, ^31^, ^32^ ^]^

To probe the synthesis kinetics, we monitored the PL intensity as a function of reaction time (Figure 3b). The luminescence intensity increases rapidly and saturates within 5 min, confirming that the VSP method enables the complete and swift conversion to the emissive MOC phase. Crucially, this rapid rise in PL intensity correlates directly with the increasing proportion of bridging sulfur atoms (A_B_/A_T_ ratio) observed in the XPS analysis (Figure S7, Supporting Information). This correlation strongly suggests that the formation of the extended 1D wire structure, facilitated by these bridging sulfur atoms, is directly responsible for creating the highly emissive states. The exciton dynamics are further investigated using time‐resolved PL measurements (Figure 3c). The PL decay map reveals a dynamic redshift of the emission peak from 596 nm (at 5 ns) to 610 nm (at 250 ns), accompanied by a lengthening of the exciton lifetime from 112.8 to 199.0 ns at longer wavelengths (Figure S8, Supporting Information). This behavior is a typical feature of STEs relaxing within a broad distribution of states. Furthermore, a large Huang–Rhys factor of 30.2 was extracted from temperature‐dependent PL measurements, quantifying the strong electron‐phonon coupling in this 1D material (Figure S9, Supporting Information). This strong electron‐phonon coupling further supports the STE model, suggesting that the previously observed broad emission and large Stokes shift are indeed the characteristic signatures of this mechanism.^[^ ^29^, ^30^, ^31^, ^32^, ^33^ ^]^ The rapid formation of these highly luminescent films motivates their integration into practical devices; therefore, we next investigated their electroluminescent performance.

**Figure 4:**
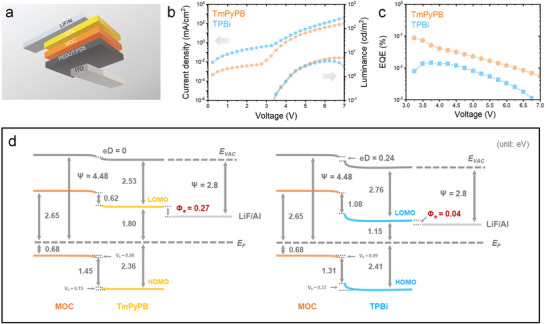
Performance and interfacial energetics of AgSPhCOOMe MOC‐based LEDs (MOCLEDs). a) Schematic of the device architecture. b) Current density–voltage–luminance (*J*–*V*–*L*) characteristics for devices using TmPyPB and TPBi as the electron transport layer (ETL). c) Corresponding external quantum efficiency (EQE) vs current density. d) Energy level diagrams of MOC/ETL interfaces constructed from photoelectron spectroscopy data. (E_VAC_, E_F_, V_b_, eD, and Ψ denote the vacuum level, Fermi level, band bending, interface dipole, and work function, respectively.).

To convert the excellent photoluminescence of the VSP‐grown films into electroluminescence, we fabricated multilayer MOCLEDs using the device architecture depicted in **Figure** **4**a. In this structure, the key AgSPhCOOMe emissive layer was formed directly onto a PEDOT: PSS hole‐transport layer via our VSP method. To complete the device and systematically investigate the impact of charge transport on performance, we utilized two different materials, TmPyPB and TPBi, as the electron‐transport layer (ETL) before depositing the final LiF/Al cathode.

The fabricated MOCLEDs generate broadband electroluminescence that spans the visible to near‐infrared (NIR) region, with a FWHM of 142 nm (Figure S10, Supporting Information). This broad NIR emission is particularly valuable as it falls within the transparency window of biological tissues. This characteristic makes these 1D MOCs a promising material for bio‐imaging and photodynamic therapy applications.^[^ ^34^, ^35^, ^36^, ^37^ ^]^

The performance of the MOCLEDs was evaluated to understand the impact of the different ETLs. Figure 4b displays the current density–voltage–luminance (*J*–*V*–*L*) characteristics of the two device types. The TPBi‐based device exhibits a typical characteristic of charge injection imbalance: a significantly higher current density and yet lower maximum luminance compared to its TmPyPB counterpart. This suggests that a large portion of injected carriers traverse the TPBi device without recombining. Conversely, the lower current density coupled with higher luminance in the TmPyPB device indicates a more balanced charge population and, consequently, a more efficient conversion of injected carriers into photons. This difference in charge‐to‐photon conversion efficiency is quantified by the external quantum efficiency (EQE) measurements shown in Figure 4c. The TmPyPB‐based MOCLED demonstrates a substantially higher EQE across the entire operating range, reaching a maximum value of 0.09%. This is more than six times higher than the peak EQE of 0.014% achieved by the TPBi‐based counterpart. To elucidate the origins of this significant performance difference, we investigated the electronic structures at the MOC/ETL interfaces in detail using in situ UPS.^[^ ^38^, ^39^, ^40^ ^]^

The resulting energy level diagrams,^[^ ^41^ ^]^ presented in Figure 4d, reveal that the stark difference in device performance can be traced to the interfacial energetics at the MOC/ETL junction^[^ ^40^ ^]^ (UPS data are shown in Figures S11 and S12, Supporting information). A key distinction appears at the MOC/TPBi interface, where a significant interface dipole (eD) of 0.24 eV is formed, whereas no such eD is detected for the MOC/TmPyPB interface. This eD creates an additional potential step that impedes electron transport, compounding the difficulty of charge injection specifically for the TPBi device. Beneficially for both device configurations, MOC‐TmPyPB and MOC‐TPBi provide considerable hole‐blocking barriers against the MOC (1.45 and 1.31 eV, respectively), which effectively confine holes within the emissive layer to promote recombination. However, the most critical factor governing the overall efficiency difference is the electron injection barrier. The barrier from TmPyPB to the MOC is 0.62 eV, substantially lower than the 1.08 eV barrier from TPBi. This lower electron injection barrier in the TmPyPB device alleviates the charge injection imbalance discussed previously, facilitating more balanced electron‐hole populations within the emissive layer and thus leading to the observed enhancement in EQE.^[^ ^41^, ^42^ ^]^

While this work demonstrates a significant breakthrough in realizing the first MOCLEDs, the results also highlight that suboptimal charge carrier management remains a key limitation to achieving higher efficiencies. The electron injection barrier, even in the optimized device, is still considerable (0.62 eV). Looking forward, unlocking the full potential of MOCs as high‐performance light emitters will require the broader research community to address several fundamental challenges (Table S2, Supporting Information). These include further tuning of MOC electronic structures through new ligand designs, developing effective *n*‐ and *p*‐type doping strategies, and understanding and passivating electronic defects,^[^ ^43^, ^44^ ^]^ such as anti‐site defects or vacancies that can impede charge transport and recombination. Overcoming these material‐level hurdles will be crucial for the practical application of MOCLEDs in next‐generation lighting and display technologies.

## 3 Conclusion

In summary, we have overcome the critical challenges of slow synthesis and the complete absence of electroluminescent devices in the field of metal–organic chalcogenides (MOCs). We developed a facile and rapid vapor‐assisted solution process (VSP) that fabricates highly luminescent (PLQY > 37%), 1D AgSPhCOOMe films in under 5 min at ambient pressure. This robust, vacuum‐free synthesis platform enabled the successful integration of these materials into light‐emitting diodes (MOCLEDs), demonstrating electroluminescence from this material class. The optimized device, using TmPyPB as an electron transport layer, exhibited broadband electroluminescence and a peak external quantum efficiency (EQE) of ≈0.1%. Our detailed analysis traced this success to favorable interfacial energetics, particularly the lower electron injection barrier provided by TmPyPB. However, this same investigation also revealed that a considerable electron injection barrier (0.62 eV) persists, pinpointing it as the primary bottleneck fundamentally limiting the current device efficiency. This finding highlights a clear and critical path for future research: focused efforts on developing new ligands, effective doping strategies, and advanced interface engineering are essential to resolve this carrier injection challenge. By addressing these next‐generation materials engineering hurdles, the full potential of MOCs as a new class of high‐performance, solution‐processable emitters for lighting and display technologies can finally be unlocked.

## Conflict of Interest

The authors declare no conflict of interest.

## Supporting information



Supporting Information

## Data Availability

The data that support the findings of this study are available from the corresponding author upon reasonable request.
