# Supplementary material for: Electroluminescence From a 1D Metal–Organic Chalcogenide Enabled by a Minute‐Scale Facile Synthesis
Source: Adv Sci (Weinh). 2025 Sep 24;12(47):e13328. doi: 10.1002/advs.202513328 (PMC12713059; doi:10.1002/advs.202513328)
Supplement: Supplementary file 1 — Supporting Information [file ADVS-12-e13328-s001.docx]

Supporting Information

Electroluminescence from a One-Dimensional Metal-Organic Chalcogenide Enabled by a Minute-Scale Facile Synthesis

Sang-Hyun Chin^[a]^, Daseul Lee^[b]^, Donggyu Lee^[a]^, Seunghwan Kim^[c]^, Byeongjoo Kang^[d]^, Kwanghyun Chung^[a]^, Tong-Il Kim^[a]^, Jieun Yeon^[a]^, Su Hwan Lee^[b]^, Sang Woo Bae^[b]^, Woojae Kim^[d]^, Soohyung Park^[c, e]^, Kwanpyo Kim^[a]^, Young-Hoon Kim^*,[b]^, and Yeonjin Yi^*,[a]^

[a] S.-H. Chin, D. G. Lee, K. Chung, T.-I. Kim, J. Yeon, K. Kim, Y. Yi

Department of Physics, Yonsei University, Seoul 03722, Republic of Korea

E-mail: yeonjin@yonsei.ac.kr

[b] D. S. Lee, S. H. Lee, S. W. Bae, Y.-H. Kim

Department of Energy Engineering, Hanyang University, Seoul 04763, Republic of Korea

E-mail: younghoonkim@hanyang.ac.kr

[c] S. Kim, S. Park

Advanced Analysis Center, Korea Institute of Science and Technology (KIST), Seoul 02792, Republic of Korea

[d] B. Kang, W. Kim

Department of Chemistry, Yonsei University, Seoul 03722, Republic of Korea

[e] S. Park

Division of Nano & Information Technology, KIST School, University of Science and Technology (UST), Seoul 02792, Republic of Korea.

**Experimental Section**

***Materials*:** Methyl thiosalicylate (97 %), acetonitrile (99.8%, anhydrous) and silver(I) nitrate (99.0 %) were purchased from Sigma Aldrich. 2,2′,2″-(1,3,5-benzinetriyl)-tris(1-phenyl-1-H-benzimidazole) (TPBi) and 1,3,5-Tris(3-pyridyl-3-phenyl)benzene (TmPyPB) were purchased from EM Index. LiF and aluminum were purchased from iTASCO. All materials were used as received without further purification.

***Synthesis of AgSPhCOOMe MOCs*:** Silver(I) nitrate (AgNO_3_) solutions in acetonitrile were spin-coated onto substrates to form precursor films. These films were then placed in a sealed glass petri dish containing 50 µL of liquid methyl thiosalicylate ligand and heated on a hot plate at 130 °C for the vapor-assisted solution process (VSP).

***Fabrication of MOCLEDs*:** Patterned indium tin oxide (ITO) glass substrates substrates were sequentially cleaned by sonication in acetone and 2-isopropanol for 15 minutes each, followed by boiling in 2-isopropanol. Prior to use, the substrates were treated with UV–ozone for 10 minutes. A hole-injection layer poly(3,4-ethylenedioxythiophene)-poly(styrenesulfonate) (PEDOT:PSS) was spin-coated onto the prepared substrate and baked at 150 °C for 1 hour. Subsequently, the AgSPhCOOMe emissive layer was formed by spin-coating a 0.05 M AgNO_3_ solution in acetonitrile at 8000 rpm, followed by the VSP reaction inside a nitrogen-filled glovebox. The samples were then transferred to a glovebox-integrated vacuum thermal evaporator. A 40 nm-thick layer of either TmPyPB or TPBi was deposited to serve as the electron-transport and hole-blocking layer. Device fabrication was completed by the thermal evaporation of a LiF (1 nm) / Al (100 nm) cathode under high vacuum (~10^–7^ mbar), which defined an active area of 5 mm^2^.

***Characterization*:** The surface morphology of MOC thin films was characterized using a field-emission scanning electron microscopy (FE-SEM, 7610F-Plus, JEOL). High-resolution XRD data (*θ* − 2*θ* scans) were collected using diffractometer (Smartlab, Rigaku) in a parallel beam geometry with Cu Kα (λ = 1.5406 Å, four-bounced Ge (220) monochromatized beam) X-ray source operated at 45 kV and 30 mA. Temperature-dependent PL and PL quantum yields were evaluated using an integrating sphere-coupled spectrometer (FP-8550, JASCO). Photoelectron spectroscopy measurements were performed in an ultra-high vacuum system. Ultraviolet photoelectron spectroscopy (UPS) was conducted using a SPECS PHOIBOS 150 hemisphere analyzer and a He I_α_ (*hν* = 21.22 eV) UV source with monochromator. A sample bias of -5 V was applied for secondary electron cutoff (SECO) measurements. Inverse photoelectron spectroscopy (IPES) was performed in isochromat mode using a low-energy electron gun and a bandpass filter (SrF_2_-NaCl combination) with a pass energy of 9.5 eV. The current density-voltage-luminance (J-V-L) characteristics of MOCLEDs were measured using a Keithley 2400 source measurement unit and a Minolta CS2000 spectroradiometer. Time-resolved photoluminescence spectra were measured using an ICCD detector (PI-MAX4, Princeton Instruments) after photoexcitation by 405 nm pump beams generated from an integrated diode pumped Q-switched Nd:YAG laser and optical parametric oscillator (NT242, EKSPLA).


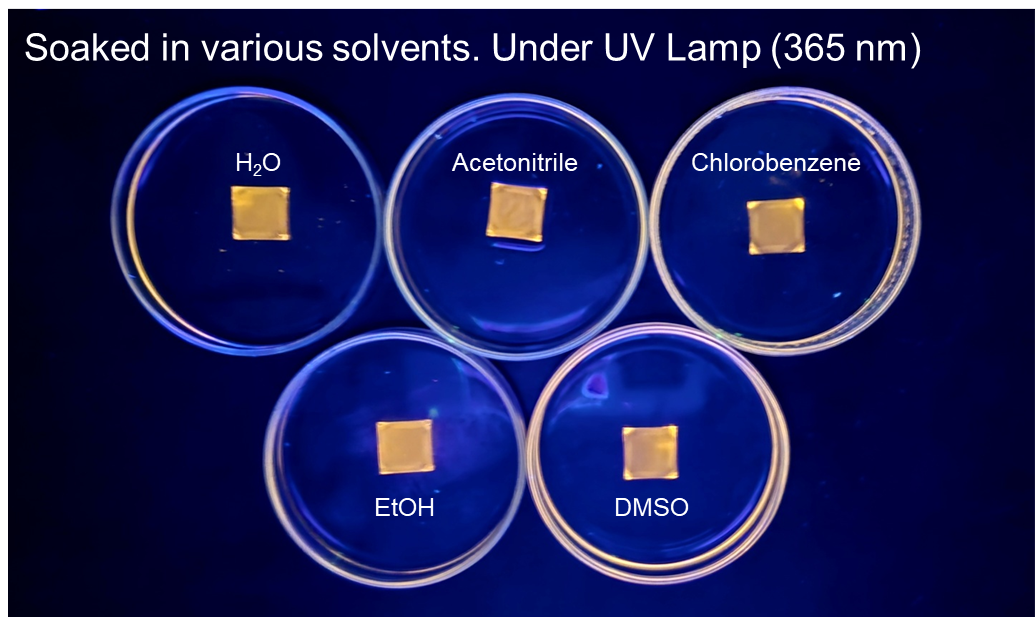


**Figure S1**. Luminescent VSP-grown AgSPhCOOMe films soaked in various solvents. (Under 365 nm UV Lamp)

**Figure S1** shows five Petri dishes containing MOC films formed on square indium-tin oxide (ITO) substrates, which are immersed in various solvents: water (H_2_O), acetonitrile, chlorobenzene, ethanol (EtOH) and dimethyl sulfoxide (DMSO). The film retains its luminescence in all solvents, which indicates its chemical stability and signifies that the soluble silver(I) nitrate precursor has been fully converted into an insoluble MOC product.


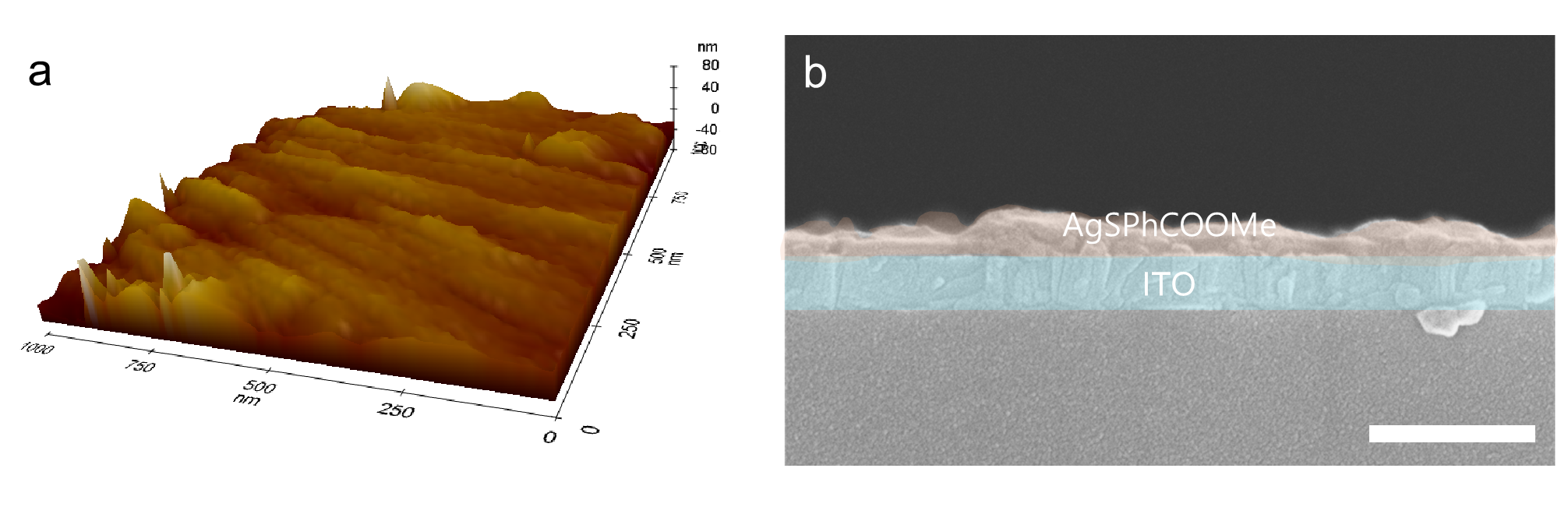


**Figure S2**. **Morphological characterization of the as-synthesized AgSPhCOOMe film on ITO substrate, demonstrating continuous and pinhole-free structures.** (a) A three-dimensional atomic force microscopy (AFM) image. (b) A cross-sectional scanning electron microscopy (SEM) image of the film on the ITO substrate (scale bar: 500 nm).

The 3D AFM image (**Figure S2a**) reveals a surface topography with a calculated root-mean-square (RMS) roughness of 11.049 nm. However, the cross-sectional SEM image (**Figure S2b**) provides more crucial information regarding the film's integrity. Despite the notable surface roughness, the image clearly demonstrates that the AgSPhCOOMe layer forms a continuous and compact film, uniformly covering the entire ITO substrate. No discernible pinholes, voids, or discontinuities that would expose the underlying ITO were observed.

**
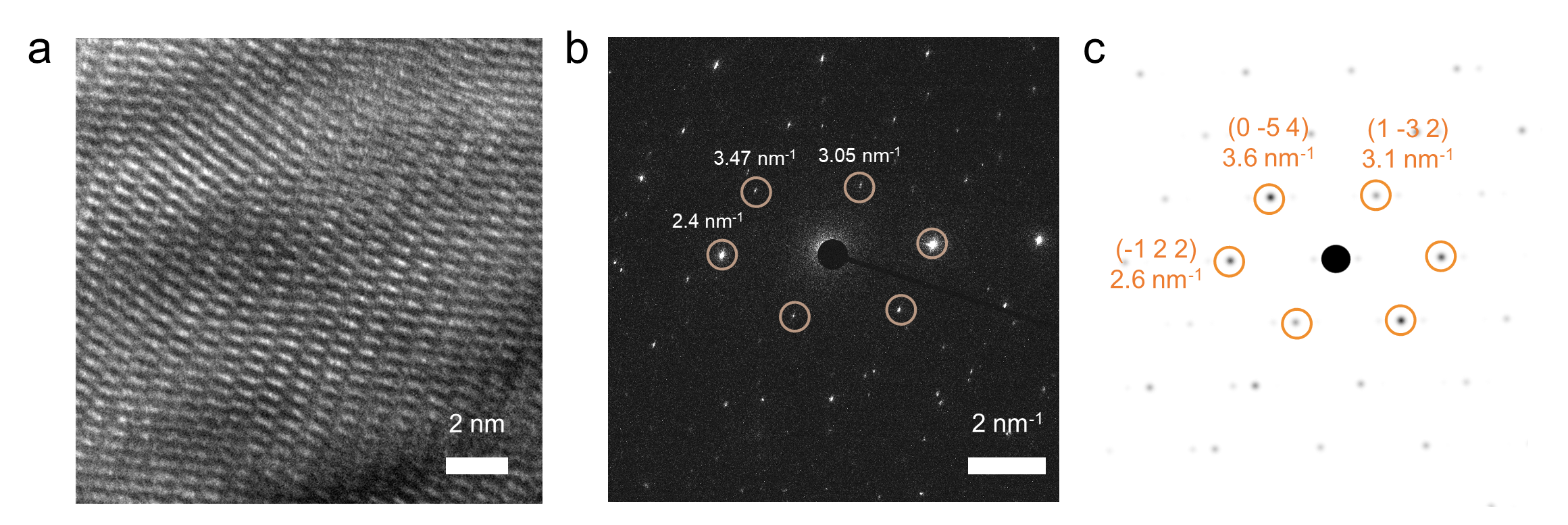
**

**Figure S3**. **Structural analysis with transmission electron microscopy (TEM).** (a) High-resolution TEM (HRTEM) image of AgSPhCOOMe. (b) Selected area diffraction pattern (SAED) of AgSPhCOOMe. (c) Simulated diffraction pattern of AgSPhCOOMe.

**Figure S3** illustrates the structural analysis of AgSPhCOOMe via TEM. The HRTEM image in **Figure S3a** confirms the 1D-growth of AgSPhCOOMe, consistent with the main text (**Figure 1**). **Figure S3b** displays a clear spot diffraction pattern, indicative of the highly crystalline nature of the VSP-grown AgSPhCOOMe. To index these spots, a simulated pattern in **Figure S3c** was generated based on a proposed monoclinic structure. Excellent agreement between the experimental and simulated patterns was achieved when the simulation was tilted by approximately 5 degrees off a major zone axis. Notably, while the underlying structure of AgSPhCOOMe is monoclinic, the pattern exhibits a high degree of pseudo-hexagonal symmetry. Furthermore, the sharp and regularly arranged diffraction spots confirm the high crystallinity and structural integrity of the analyzed domain.


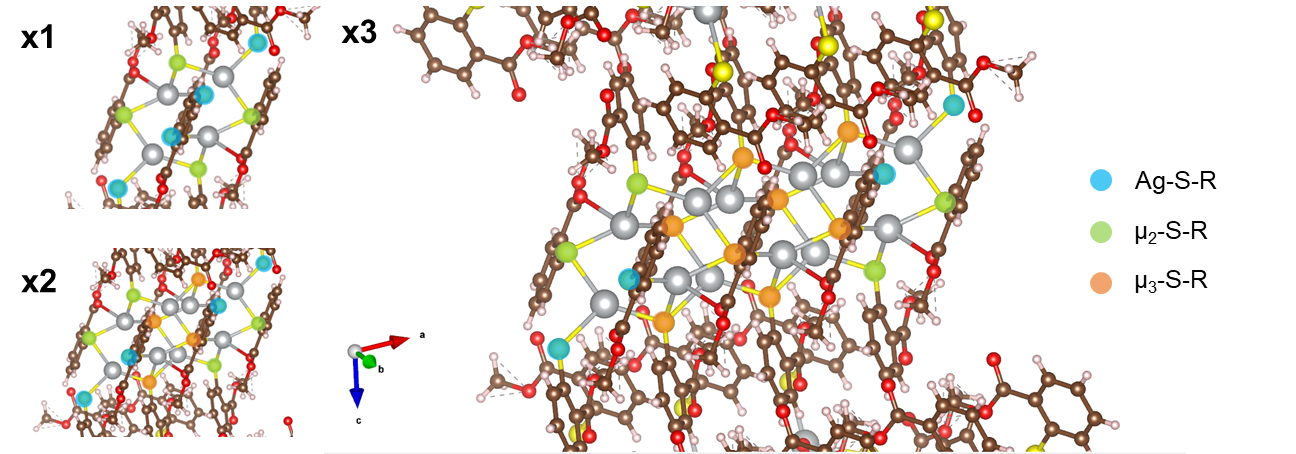


**Figure S4**. Atomic visualization of distinct sulfur coordination environments in the 1D AgSPhCOOMe structure.

**Figure S4** illustrates the different structural roles of sulfur atoms corresponding to the components observed in the S 2p XPS spectra (main text, **Figure 2c-e**). The coordination environments are categorized as follows:

1. Terminal Sulfur (A_T_): Located at the chain-ends, where a sulfur atom is bonded to a single Ag atom, represented by blue spheres.
2. Bridging Sulfur (A_B_): Forms the internal backbone of the 1D chain by linking multiple Ag atoms. This category is further divided into:

μ_2_-S-R: Sulfur bonded to two Ag atoms (represented by green spheres).

μ_3_-S-R: Sulfur bonded to three Ag atoms (represented by red spheres).

This visualization clarifies the physical meaning of the XPS analysis discussed in the main text: the progressive increase in the proportion of bridging sulfur (A_B_) directly corresponds to the elongation of the 1D chains.


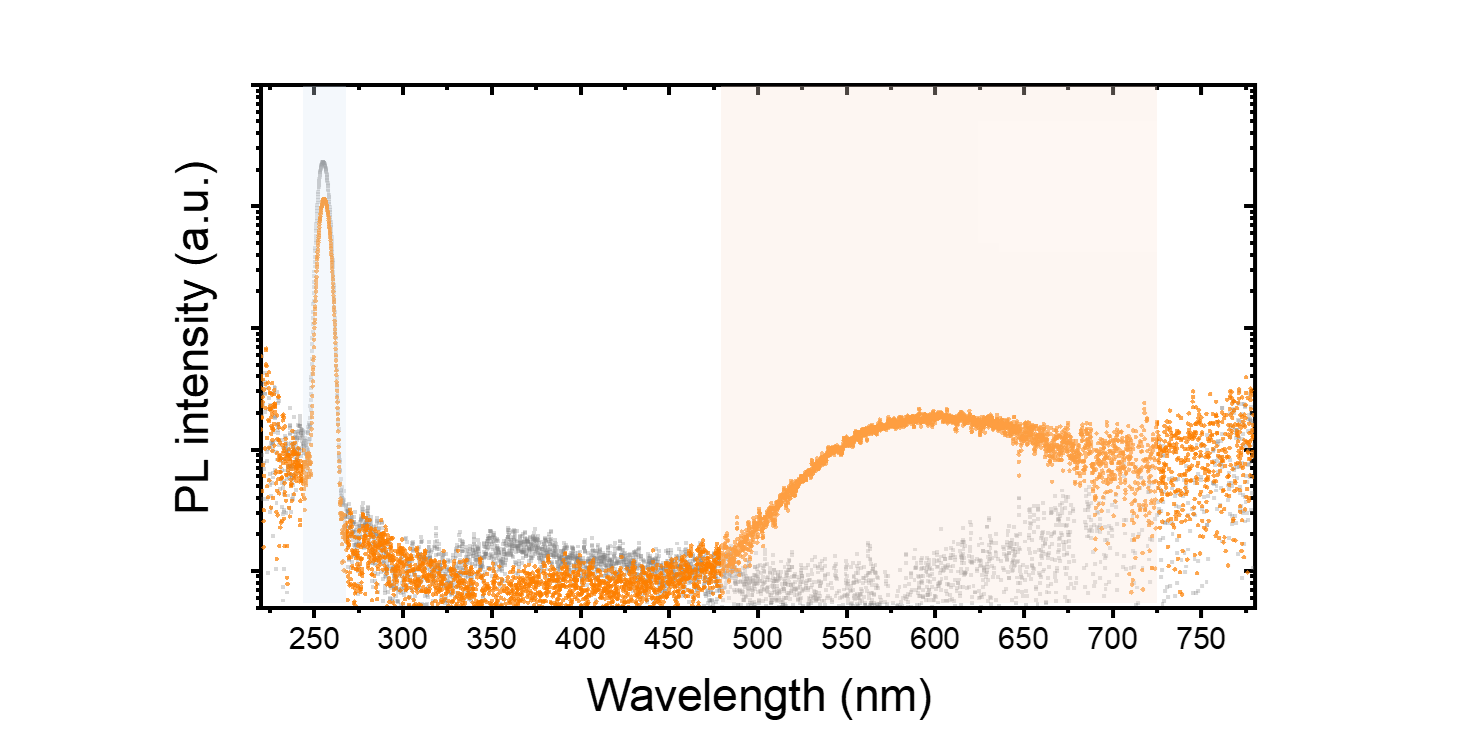


**Figure S5**. Photoluminescence quantum yield (PLQY) measurement of a VSP-grown AgSPhCOOMe film.

The PLQY was determined using an integrating sphere with an excitation wavelength of 256 nm. The plot displays the raw spectral data used for the calculation. The grey curve represents the spectrum of the excitation laser scattered from a blank substrate (reference), while the orange curve shows the spectrum from the AgSPhCOOMe film, which includes both the attenuated laser scattering and the PL signal of the film. By integrating the light absorbed by the film and the light emitted via photoluminescence from these two spectra, the PLQY is calculated to be 37.5%.

**
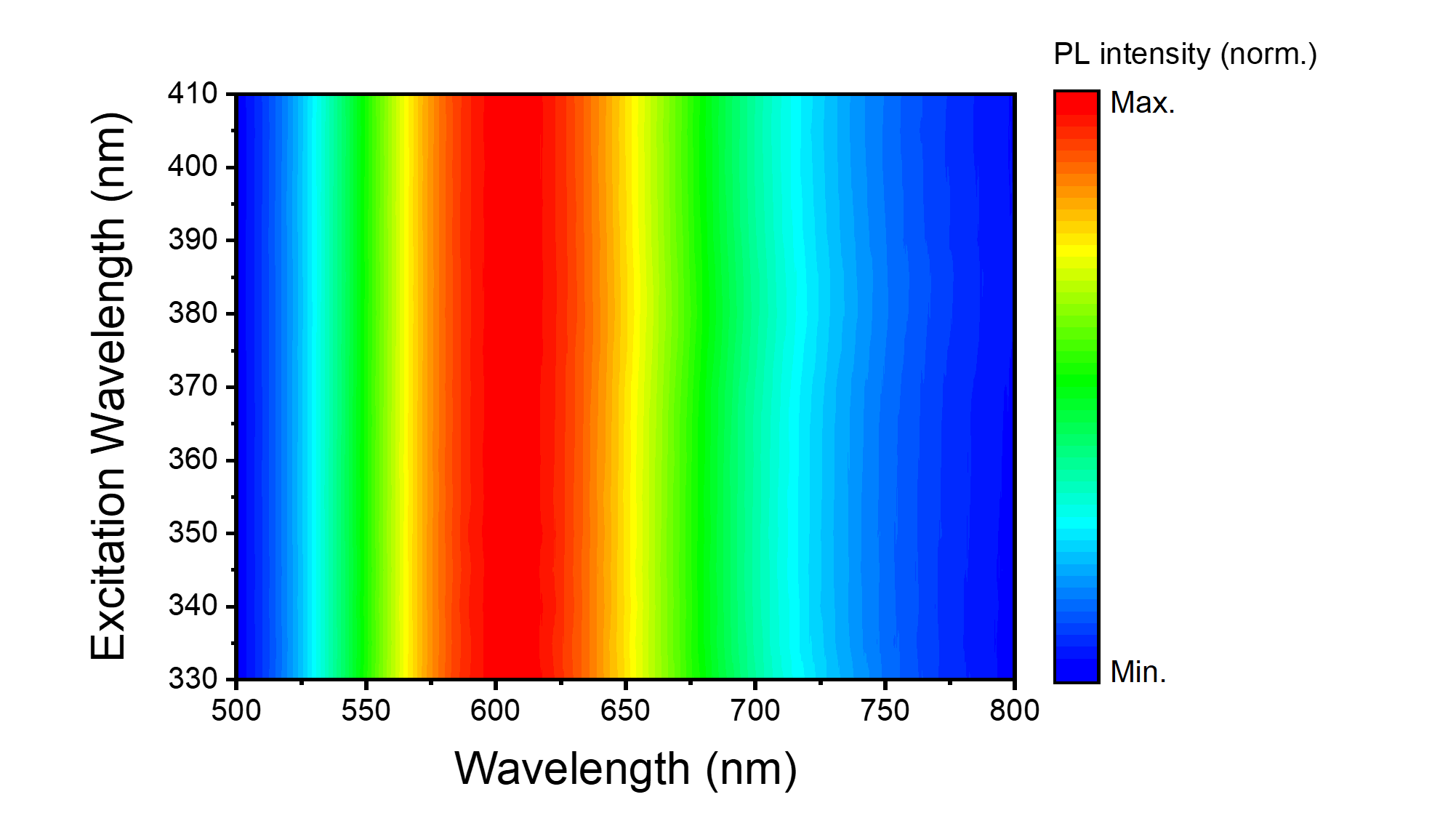
**

**Figure S6**. Contour map of the normalized photoluminescence (PL) intensity for the VSP-grown AgSPhCOOMe film..

To investigate the origin of the emission, the photoluminescence intensity was mapped as a function of both excitation and emission wavelengths in **Figure S6**. A key feature revealed in this map is that the emission maximum remains constant at approximately 600 nm across the entire excitation range studied, from 330 nm (above the bandgap) to 410 nm (near the excitonic peak). This independence of the emission profile from the excitation energy is a characteristic signature of luminescence originating from a single, relaxed excited state. This provides strong corroborating evidence that the emission proceeds via a self-trapped exciton (STE) mechanism.

**
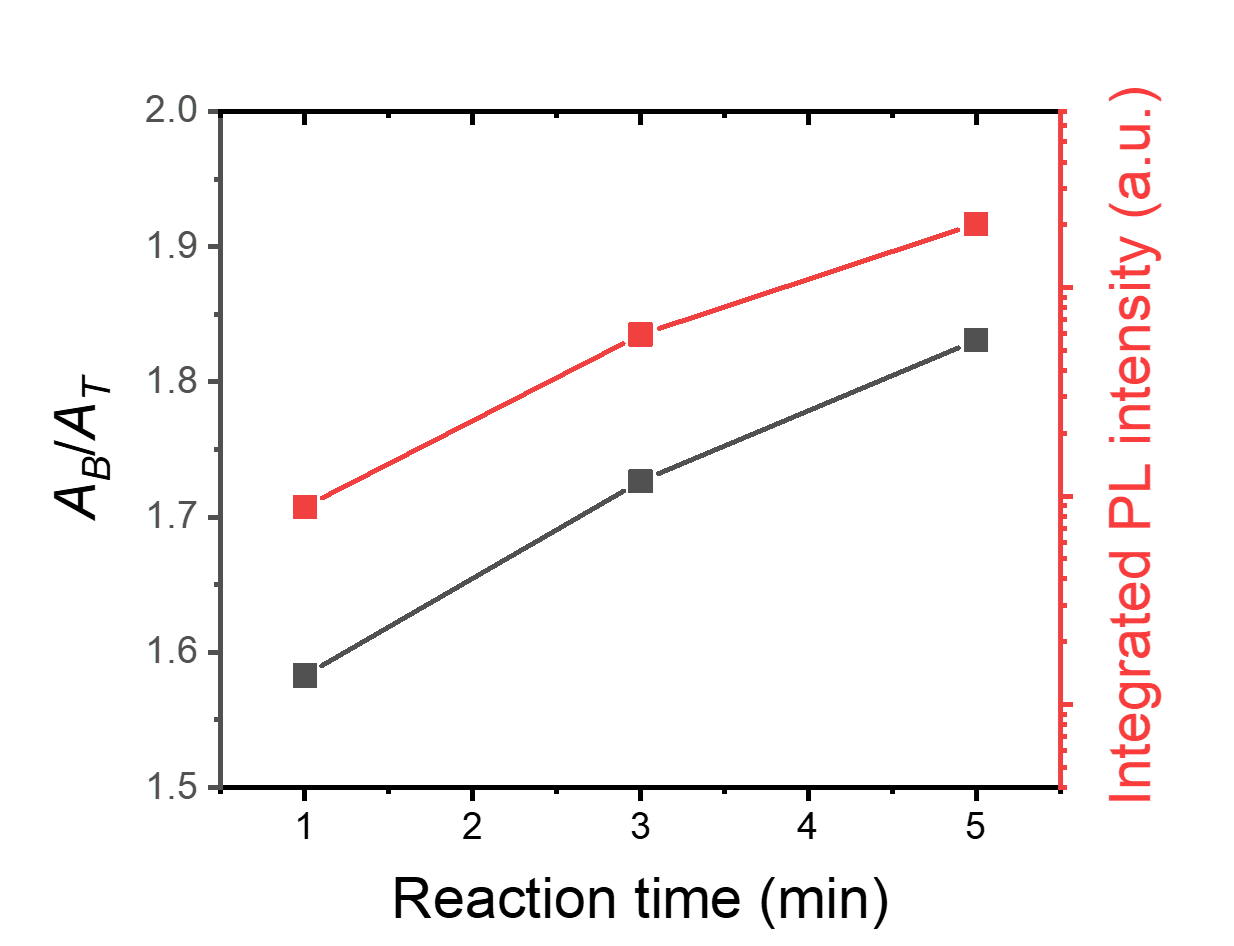
**

**Figure S7**. Correlation between Structural Evolution (**A_B_/A_T_** ratio) and PL intensity during VSP reaction.

**Figure S7** directly correlates the structural evolution of the MOC film with its emergent optical properties during the initial 5 minutes of the VSP reaction.

The black data points represent the bridging-to-terminal sulfur area ratio (**A_B_/A_T_**), as determined from the S 2p XPS spectra in the main text (**Figure 2**). This ratio progressively increases with reaction time, signifying the continuous elongation of the 1D chains as more sulfur atoms form the internal backbone. The red data points represent the integrated PL intensity measured from the same samples. The PL intensity rapidly increases and saturates in the same 5-minute timeframe.

The strong positive correlation between these two trends provides clear evidence that the formation and elongation of the 1D wire structure is directly responsible for the development of the material's highly emissive states.


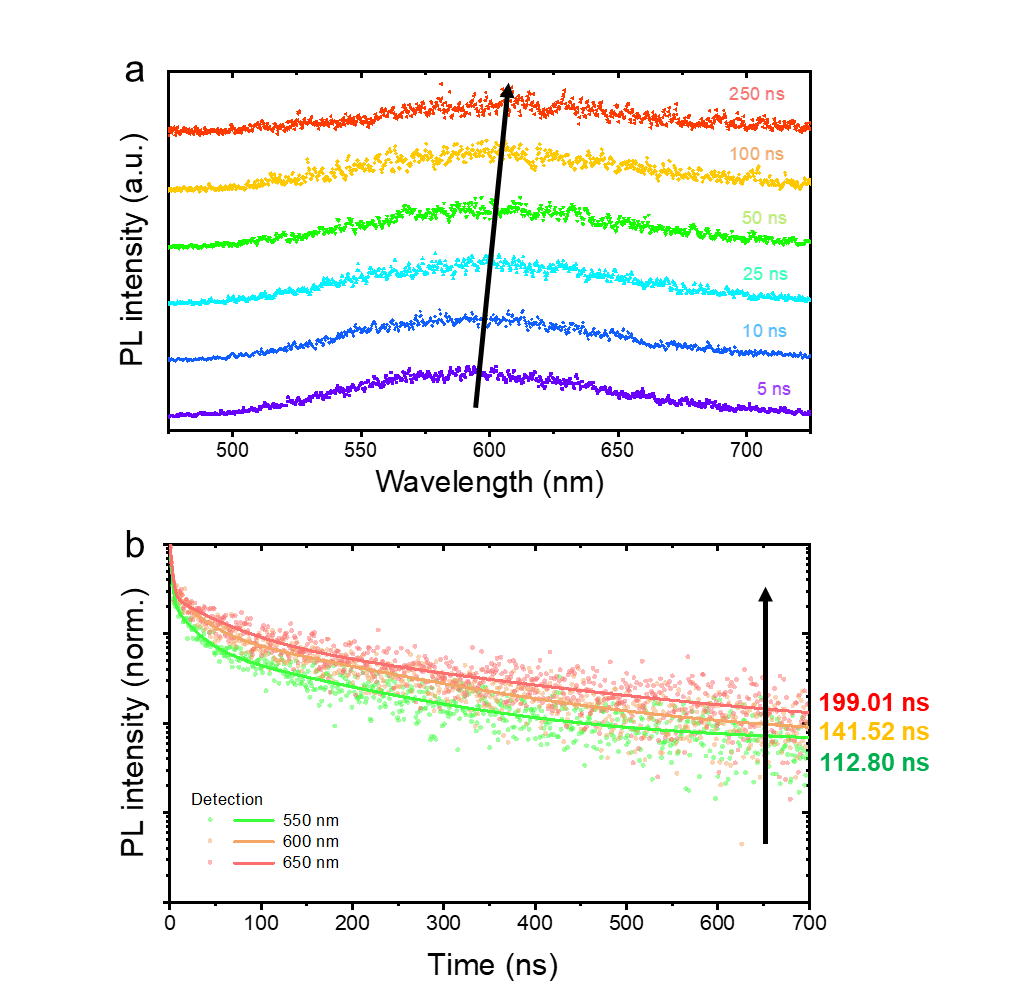


**Figure S8**. Time-resolved photoluminescence (PL) dynamics of the VSP-grown AgSPhCOOMe film.

**Figure S8** provides detailed data on the exciton dynamics of the film. Panel (a) displays a series of PL spectra recorded at different delay times after pulsed excitation. The emission peak exhibits a clear dynamic redshift, shifting from 596 nm at 5 ns to 610 nm at 250 ns. Panel (b) shows the corresponding normalized PL decay traces monitored at various emission wavelengths. Using multiexponential fitting, we extracted time constants of the PL decay kinetics and calculated their weighted average to determine the mean exciton lifetime. The exciton lifetime is observed to lengthen at longer wavelengths, with representative lifetimes determined to be 112.8 ns (green), 141.5 ns (orange), and 199.0 ns (red). Taken together, the dynamic redshift of the emission and the wavelength-dependent lifetimes are characteristic features of self-trapped excitons (STEs) relaxing within a broad manifold of states.


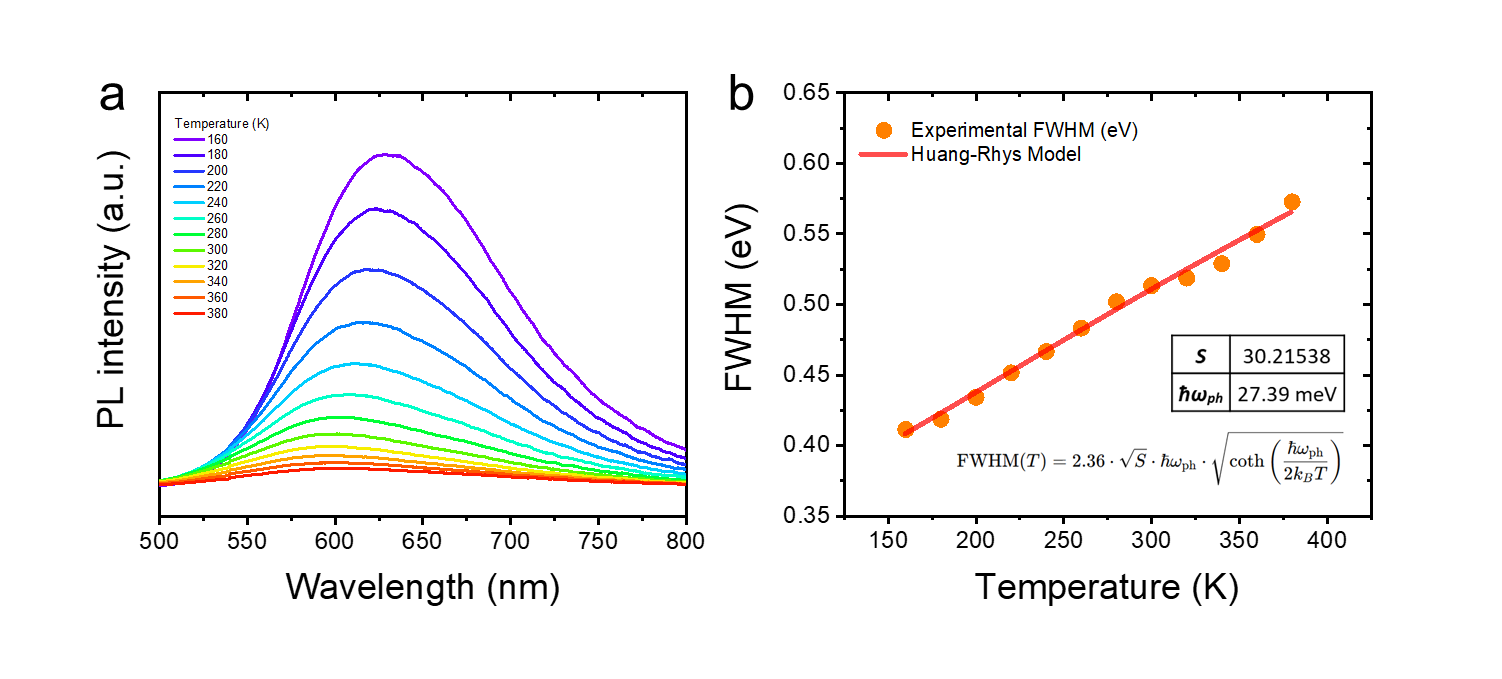


**Figure S9**. Analysis of electron-phonon coupling via temperature-dependent photoluminescence (PL) spectroscopy.

To quantify the strength of electron-phonon coupling in the AgSPhCOOMe film, we measured temperature-dependent PL spectra.

(a) PL spectra recorded at various temperatures ranging from 160 K to 380 K. As is common for semiconducting materials, the overall PL intensity decreases with increasing temperature due to the thermal activation of non-radiative recombination pathways.

(b) The temperature dependence of the emission's full width at half maximum (orange circles) is fitted using the Huang-Rhys model (red line). This analysis yields a large Huang-Rhys factor (S) of 30.2 and an average interacting phonon energy (*ħω*_ph_) of 27.4 meV.

The large S factor provides a quantitative confirmation of the strong electron-phonon coupling in this material. This is a prerequisite for the formation of self-trapped excitons (STEs) and strongly supports the emission mechanism discussed in the main text.


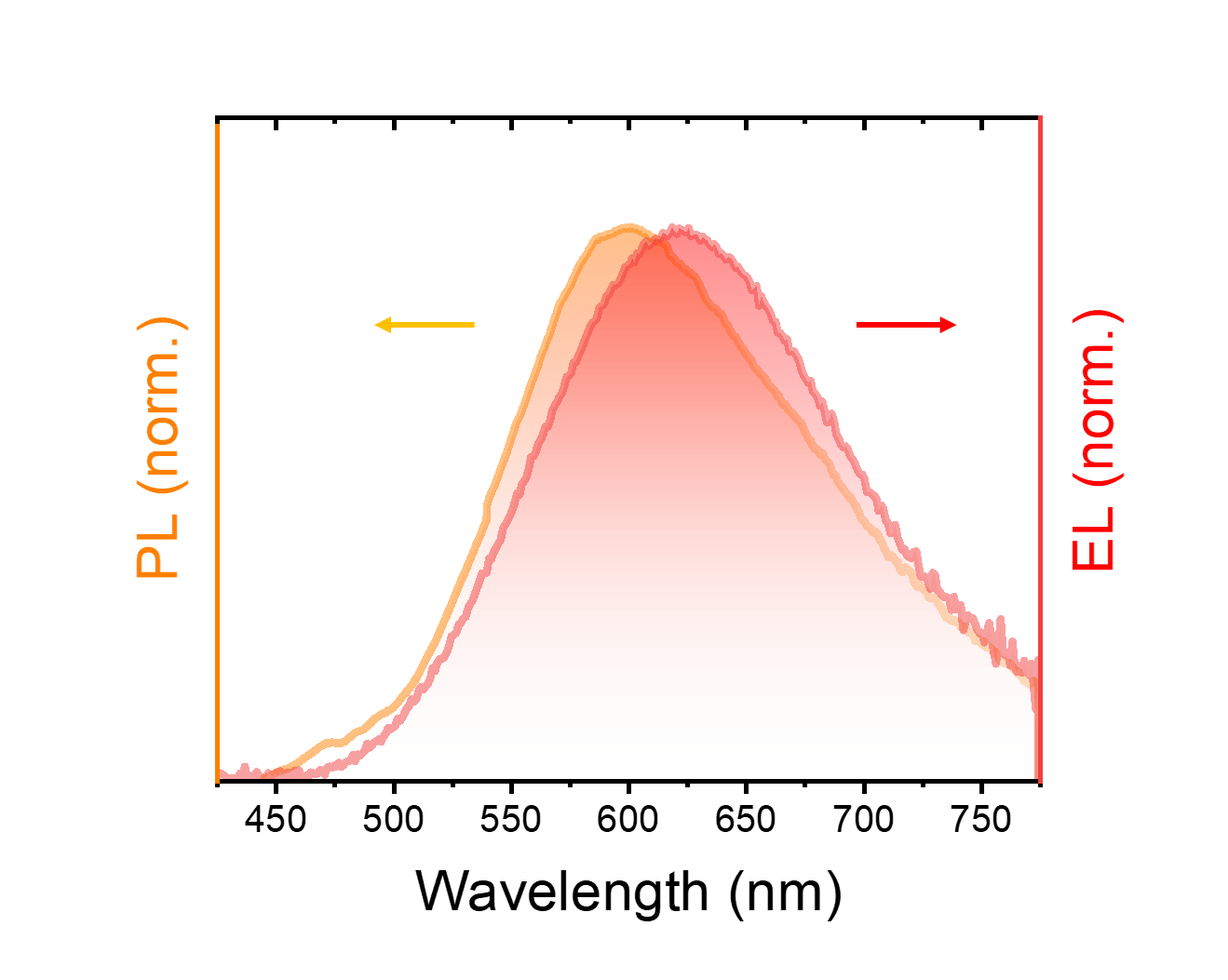


**Figure S10**. Comparison of Photoluminescence (PL) and Electroluminescence (EL) Spectra.

**Figure S10** compares the normalized PL spectrum (orange), generated via photoexcitation, with the EL spectrum (red), generated from a fabricated MOCLED. Both spectra show nearly identical broadband emission profiles in the visible to near-infrared (NIR) region, with peaks centered around 600 nm. The EL spectrum has a full width at half maximum (FWHM) of 142 nm.

The strong spectral overlap is clear evidence that the same emissive states—the self-trapped excitons (STEs) discussed in the main text—are populated under both optical and electrical excitation. The broad emission, spanning the deep-red to NIR region, is particularly valuable for potential applications such as bio-imaging and photodynamic therapy, as it falls within the transparency window of biological tissues.


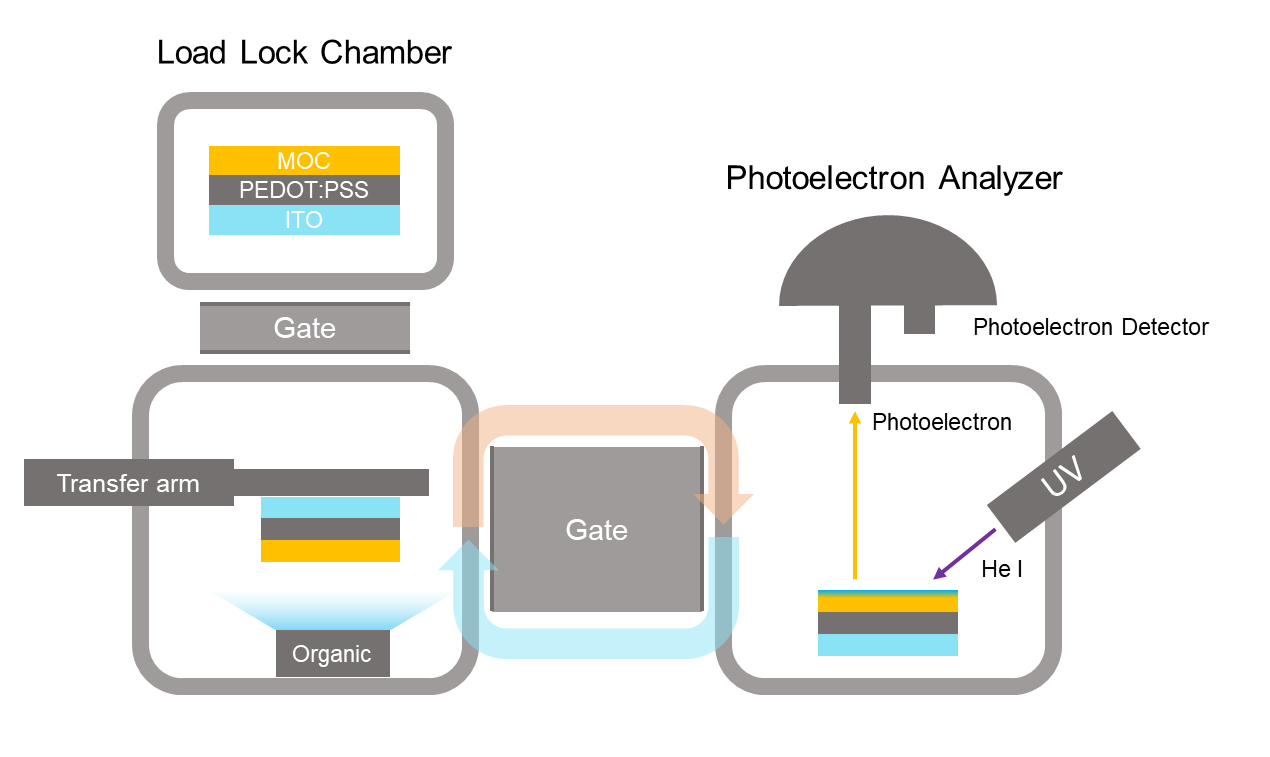


**Figure S11**. Schematic of the interconnected in-situ deposition and photoelectron spectroscopy (PES) system.

**Figure S11** illustrates the experimental setup used to investigate the MOC/ETL interfaces without exposing the sample to ambient conditions. The process involves several steps performed under high vacuum:

1. A substrate with the completed MOC layer is introduced into the system via a load-lock chamber.
2. In an interconnected deposition chamber, a thin layer of an organic electron-transport material (ETL) is deposited onto the MOC surface using a thermal evaporation source.
3. The sample is then transferred through a gate valve to the main analysis chamber and the UPS measurements are performed.

This in-situ, layer-by-layer deposition and measurement sequence is repeated to precisely track the evolution of the electronic structure at the interface, allowing for the determination of parameters such as band bending (V_b_) and the interface dipole (eD), as discussed in the main text.


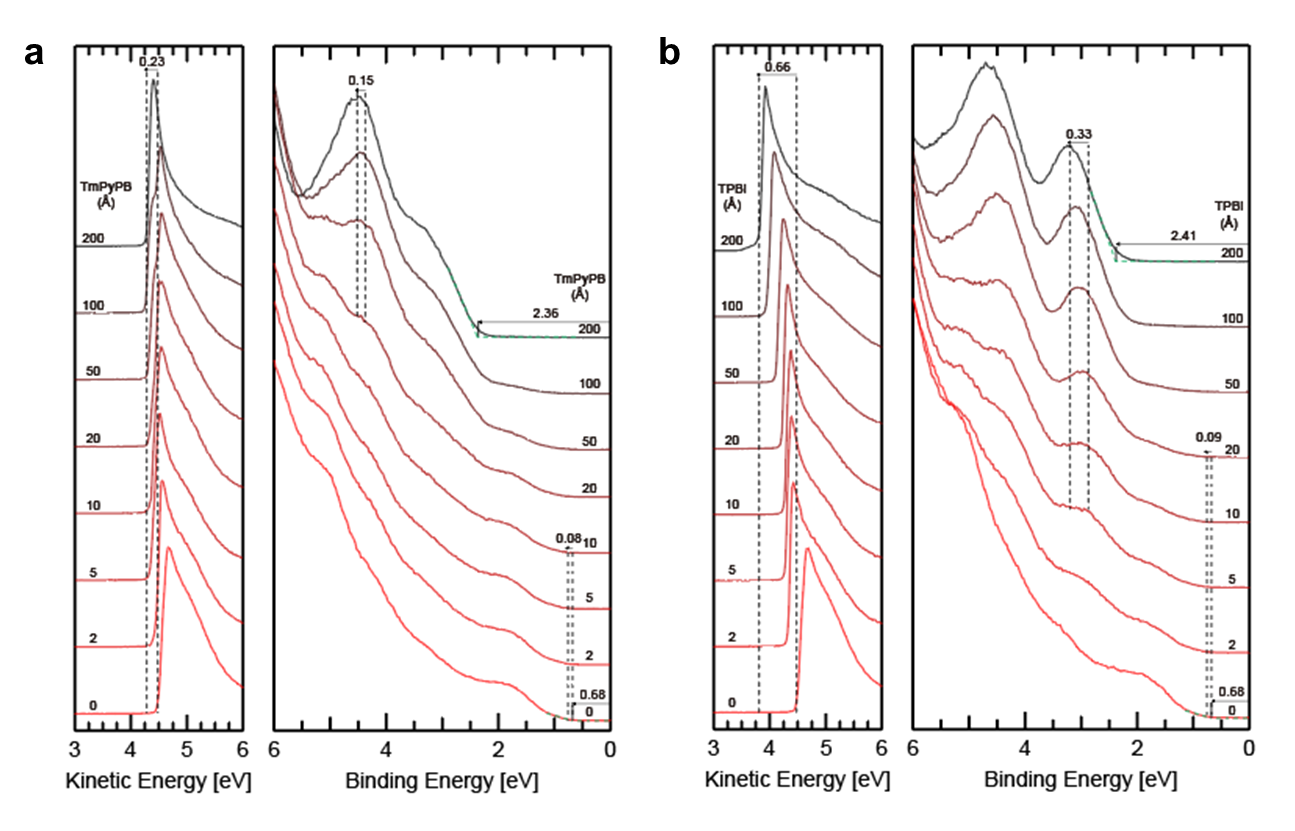


**Figure S12**. Evolution of ultraviolet photoelectron spectroscopy (UPS) spectra during the *in-situ* formation of the MOC/ETL interfaces.

**Figure S12** presents the detailed UPS data used to determine the interfacial energetics between the MOC film and two different electron transport layers (ETLs). The spectra show the evolution of the (left panels) secondary electron cutoff (SECO) and (right panels) valence band (VB) regions as the ETL is incrementally deposited onto the MOC film. This process was performed for two systems, (a) MOC/TmPyPB and (b) MOC/TPBi.

For both systems, spectra were recorded for the pristine MOC surface and after the sequential deposition of the ETL with increasing thickness (2, 5, 10, 20, 50, 100, and 200 Å). The shifts in the SECO and VB features as a function of ETL thickness are used to calculate the vacuum level shift (and thus the interface dipole, eD) and the valence band offset, respectively. These parameters are essential for constructing the final energy level alignment diagrams shown in **Figure 4d** of the main text.

**Table S1.** Photoluminescent properties of metal organic chalcogenide coordination polymers reported to date.

| Chemical  Species | Form | Authors | Publication | PL  peak  (nm) | PL  QY  (%) | Time Duration  For Synthesis |
| --- | --- | --- | --- | --- | --- | --- |
| AgSePh | Film | W. S. Lee *et al.* | ACS Nano  2022, 16, 20318 | 467 | 0.05 | 100 °C for 3 days |
| AgTePh | Film | W. S. Lee *et al.* | ACS Nano  2022, 16, 20318 | 607 | 0.85 | 100 °C for 3 days |
| AgSePh-F_2_(2,6) | Dispersed | T. Sakurada *et al.* | J. Am Chem. Soc.  2023, 145, 5183 | 574 | 2.4 | 100 °C for 3 days |
| Cu(o-SPhCO_2_Me) | Powder | A. Abdallah *et al.* | iScience 2023, 26,  106016 | 596 | 5 | 120 °C for 24 hours |
| Ag(o-SPhCOOMe) | Dispersion | M. Aleksich *et al.* | Adv. Funct. Mater.  2025, 35, 2414914. | 579 | 22 | 80 °C for 24 hours |
| Ag(m-SPhCOOMe) | Dispersion | M. Aleksich *et al.* | Adv. Funct. Mater.  2025, 35, 2414914. | 589 | 1 | 80 °C for 5 days |
| **Ag(o-SPhCOOMe)** | **Film** | **S.-H. Chin *et al.*** | **This Work** | **601** | **37.5** | **130 °C for 5 minutes** |

**Table S2.** Key milestones in the development of room-temperature operational organic and perovskite LEDs.

| Type of LEDs | Authors | Publication | Max.  Luminance  (cd/m^2^) | External Quantum Efficiency (%) | Note |
| --- | --- | --- | --- | --- | --- |
| Organic LEDs | W. Helfrich &  W. G. Schneider | Phys. Rev. Lett.  1965, 14, 229. | N/A | N/A | Faint emission of Electroluminescent 5 mm-thick Anthracene |
| Organic LEDs | C. W. Tang &  S. A. van Slyke | Appl. Phys. Lett.  1987, 51, 913 | >1,000 | 1 | First practical OLEDs based on small molecules |
| Organic LEDs | J. H. Burroughes *et al.* | Nature  1990, 347, 539 | N/A | 0.05 | First practical OLEDs based on conjugated polymers |
| Organic LEDs | M. A. Baldo *et al.* | Nature  1998, 395, 151 | >100 | 4 | First phosphorescent OLEDs |
| Organic LEDs | C. Adachi *et al.* | J. Appl. Phys.  2001, 90, 5048 | N/A | 19 | Efficient phosphorescent OLEDs |
| Organic LEDs | Y.-S. Park *et al.* | Adv. Funct. Mater. 2013, 23, 4914 | >20,000 | 29.1 | Efficient exciplex host-assisted phosphorescent OLEDs |
| Perovskite  LEDs | L. C. Schmidt *et al.* | J. Am. Chem. Soc.  2014, 136, 850 | >0.4 | N/A | First perovskite nanocrystal LEDs |
| Perovskite  LEDs | Z.-K. Tan *et al.* | Nat. Nanotech.  2014, 9, 687 | 364 | 0.1 | First polycrystalline perovskite LEDs |
| Perovskite  LEDs | Y.-H. Kim *et al.* | Adv. Mater.  2015, 27, 1248 | 417 | 0.125 | Multicolor emissive polycrystalline perovskite LEDs |
| Perovskite  LEDs | H. Cho *et al.* | Science  2015, 350, 1222 | >10,000 | 8.53 | First practical perovskite LEDs |
| Perovskite  LEDs | Y.-H. Kim *et al.* | Nat. Photon.  2021, 15, 148 | >10,000 | 23.4 | Efficient perovskite nanocrystal LEDs |
| Perovskite  LEDs | J. S. Kim *et al.* | Nature  2022, 611, 688 | >470,000 | 28.9 | Highly efficient polycrystalline perovskite LEDs |
| **MOC**  **LEDs** | **S.-H. Chin *et al.*** | **This Work** | **5** | **0.09** | **First MOCLEDs** |
